# *egc* Superantigens Impair Monocytes/Macrophages Inducing Cell Death and Inefficient Activation

**DOI:** 10.3389/fimmu.2019.03008

**Published:** 2020-01-15

**Authors:** Sofia Noli Truant, Mauricio C. De Marzi, María B. Sarratea, María B. Antonoglou, Ana P. Meo, Laura V. Iannantuono López, María J. Fernández Lynch, Marcos Todone, Emilio L. Malchiodi, Marisa M. Fernández

**Affiliations:** ^1^Cátedra de Inmunología, Departamento de Microbiología, Inmunología, Biotecnología y Genética, Facultad de Farmacia y Bioquímica, Universidad de Buenos Aires, Buenos Aires, Argentina; ^2^Instituto de Estudios de la Inmunidad Humoral “Prof. Ricardo A. Margni” (IDEHU), UBA-CONICET, Universidad de Buenos Aires, Buenos Aires, Argentina; ^3^Departamento de Ciencias Básicas, Universidad Nacional de Luján, Luján, Argentina; ^4^Instituto de Ecología y Desarrollo Sustentable (INEDES), UNLU-CONICET, Universidad Nacional de Luján, Luján, Argentina; ^5^Hospital Dr. J. M. Ramos Mejía, Buenos Aires, Argentina

**Keywords:** superantigens, monocytes, innate immune response, gp130, MHC-II, THP-1

## Abstract

Bacterial superantigens (SAgs) are enterotoxins that bind to MHC-II and TCR molecules, activating as much as 20% of the T cell population and promoting a cytokine storm which enhances susceptibility to endotoxic shock, causing immunosuppression, and hindering the immune response against bacterial infection. Since monocytes/macrophages are one of the first cells SAgs find in infected host and considering the effect these cells have on directing the immune response, here, we investigated the effect of four non-classical SAgs of the staphylococcal *egc* operon, namely, SEG, SEI, SEO, and SEM on monocytic–macrophagic cells, in the absence of T cells. We also analyzed the molecular targets on APCs which could mediate SAg effects. We found that *egc* SAgs depleted the pool of innate immune effector cells and induced an inefficient activation of monocytic–macrophagic cells, driving the immune response to an impaired proinflammatory profile, which could be mediated directly or indirectly by interactions with MHC class II. In addition, performing surface plasmon resonance assays, we demonstrated that non-classical SAgs bind the gp130 molecule, which is also present in the monocytic cell surface, among other cells.

## Introduction

*Staphylococcus aureus* is one of the major pathogens responsible for human and veterinarian diseases and life-threatening infections ranging from skin and soft tissues to toxic shock syndrome (TSS) and sepsis (1–4). The last two conditions are characterized by a non-controlled release of proinflammatory cytokines which can lead to multiple organ failure and death (5).

Many virulence factors of *S. aureus* have been described; among them, staphylococcal enterotoxins (SEs) or superantigens (SAgs) are some of the most important. These toxins can promote immunosuppression in the infected host, allowing bacterial spread and further sepsis (6–8). SAgs are not circumscribed only to *S. aureus*; other important pathogens such us *Streptococcus pyogenes* produce a broad repertoire of toxins with SAg activity. Staphylococcal and streptococcal SAgs share a common tridimensional structure and display high similarity in their amino acid sequences (9).

SAgs interact simultaneously with major histocompatibility complex class II molecules (MHC-II) on antigen-presenting cells (APC) and with the T cell receptors (TCR) on the surface of T cells, in a non-conventional way as unprocessed molecules. Due to these interactions, a cytokine storm is released, leading to TSS and host immunosuppression (10–15). In addition, SAgs are strongly associated with autoimmune diseases and food poisoning (16–21). Since SAgs are resistant to high temperature and enzymatic treatment and can act at very low concentrations, they were classified as category B priority agents by the CDC because of their potential use in bioterrorism and biological warfare.

Staphylococcal SAgs are described as classical (SEA to SEE and TSST-1) and non-classical enterotoxins (SEG to SEU) (22–25). This division between classical and non-classical SAgs is also extended to the streptococcal pyrogenic exotoxins (SPE A–C, F–H, and J and streptococcal superantigen A) and the streptococcal mitogenic exotoxin Z (SMEZ). The interaction between immunological molecular targets and classical SAgs is very well-documented (26–33). The crystallographic structures of classical SAgs in complex with the TCR and the MHC-II molecules allowed the identification of a conserved motif over the SAgs surfaces involved in the interaction. Less is known about the interaction between these receptors on the T cells or the APCs and non-classical SAgs. The crystallographic structures available, would suggest new residues over the SAg surface involved in the interaction with TCR and MHC-II molecules (34–39).

In addition, biological differences had been reported between classical and non-classical SAgs. In this regard, an important aspect is the higher stimulatory capacity that SEB and SMEZ, both classical SAgs that bind CD28 (40–44), possess over the non-classical SAgs, for which interaction with CD28 has not been studied yet. These classical SAgs, which also interact with B7.2 (42, 43) display a stimulatory capacity three orders higher compared with SEG and SEI (27, 45, 46).

The interaction between the co-receptors CD28 and B7.2 and classical SAgs as SEB, SPEA, SMEZ, and SEA has been deeply studied (40–44, 47). A conserved motif among SAgs, located at beta strand 8-alpha helix 4, distant from the TCR and MCH-II binding site, would constitute the binding region with these new ligands. The interaction with these co-receptors could allow a full T cell activation.

The glycoprotein 130 (gp130), a signal transductor of IL-6, was also described as a new classical SAg target. Only one study reported that the staphylococcal SAg SEA could bind to gp130 on the adipocytes' surface (48). There are no other publications describing this interaction and its biological significance on cells of the immune system.

SAgs, such as other virulence factors, are encoded in mobile genetic elements located in pathogenicity islands, phages, plasmids, and transposons. The genes of non-classical SAgs, SEG, SEI, SEM, SEN, and SEO belong to the enterotoxin gene cluster, *egc*, which is located in pathogenicity island 3 (18). At present, seven forms of the *egc* operon were described (49). Sometimes, strains carrying the *egc* operon are also bearers of the *seu* gene, increasing the virulence of the strain (18, 49); *seu* is harbored in the operon instead of the pseudogenes *ent1* and *ent2*, which are present in most forms of the operon. The *egc* SAgs genes are described as the most prevalent SAgs genes (50–55).

The interaction between SAgs and TCR is very well-characterized (29, 31, 38). Less is known about the outcome of SAg interaction with innate immune cells. Some studies have analyzed the interaction of bacterial SAgs with human monocytes enriched from peripheral blood (56), dendritic cells (DCs) (57–59), and murine neutrophils (60). Since monocytes/macrophages are one of the first cells SAgs found in infected hosts and considering the effect these cells have on directing the immune response, here we investigated the effect of four SAgs of the staphylococcal *egc* operon: SEG, SEI, SEO, and SEM on monocytic–macrophagic cells, in the absence of T cells, employing THP-1 cells, a human cell line which is broadly used as a model of monocytes and macrophages (61–63). We also analyzed other molecular targets on APCs which could directly or indirectly mediate *egc* SAgs effects. We found that *egc* SAgs depleted the pool of innate immune effector cells and induced an inefficient activation of monocytic–macrophagic cells, driving the immune response to an impaired proinflammatory profile, which could be mediated directly or indirectly by interactions with MHC class II. In addition, performing surface plasmon resonance (SPR), we demonstrated that non-classical *egc* SAgs bind to the gp130 molecule, which is ubiquitously expressed in immune cells.

## Materials and Methods

### Evaluation of SAg Genes in Autochthonous Strains of *S. aureus*

*S. aureus* strains were isolated from patients and characterized in the Hospital General de Agudos “J. M. Ramos Mejía.” The isolates were microbiologically identified as *S. aureus* due to their ability to coagulate citrated rabbit plasma (positive coagulase) and to produce clumping factor. Additionally, routine antibiograms were performed. The strains were isolated in LB-agar and cultured in antibiotic-free Trypticase soy broth medium with 3% N-Z amine and 1% yeast extract (64) at 37°C with moderate agitation (200 rpm) up to DO_600nm_ = 1.

DNA was isolated using the Miniprep genomic DNA isolation kit (QIAGEN, GmbH-Germany), and its integrity was checked in 0.8% agarose gel. SAg genes in the clinical isolates were evaluated by polymerase chain reaction (PCR) (45, 65–67) using specific primers for *sags*. Fc30 (35) and hsp60 were used as a positive control (SP Table 1). Positive cases were repeated and amplified using a high-fidelity polymerase (Invitrogen, Thermo Fisher Scientific Inc.). The sequencing was developed by Macrogen Inc. (Korea).

### Sequence Analysis

Nucleotide and amino acid sequences were analyzed using the ExPASy Protein Translate program (Swiss Institute of Bioinformatics). Alignments were performed with BLAST-NCBI and ClustalW. Three subclones were analyzed to ensure that no Taq Platinum Plus (Promega)-related mutations were introduced. For construction and analysis of sequence similarities and secondary structure, ESPript software was used as described elsewhere (68). The sequences were deposited at the NCBI Protein Data Bank and published with the accession numbers MK947360, MK947361, MK947362, MK947363, MK947364, MK947365, and MK947366.

### Monospecific Mouse anti-SAg Sera

Animals were manipulated following the ethical standards of the Universidad de Buenos Aires, Facultad de Farmacia y Bioquímica, where the work was carried out (Resolution No. 2349/18). Polyclonal antisera were obtained by immunization of Balb/c mice with 1 mg/ml of recombinant SEG, SEI, or SSA mixed with Freund's adjuvant. Boosts were administered on days 7, 14, and 28. Sera obtained on day 35 were diluted 10-fold and tested by ELISA and immunoblotting. Antisera were enriched in the immunoglobulin fraction by saline precipitation followed by molecular exclusion purification. The enriched fraction was treated with pepsin and then the purified F(ab)'_2_ fragment was used in the following assays to avoid cell proliferation or activation due to the interaction of mouse Fc fragments with human Fc receptors.

### Reagents and Bacterial Strains

All chemical reagents were of analytical grade and purchased from Sigma (St. Louis, MO). Restriction enzymes, Taq DNA polymerase, T4 ligase, and buffers for cloning were purchased from New England Biolabs, Inc. (Beverly, MA). Ultrapure agarose was purchased from GIBCO BRL-Life Technologies (Rockville, MD). Recombinant Human Glycoprotein 130 Fc Chimera (gp130) was purchased from R&D (671-GP, Bio-techne, Minneapolis, MN).

Biacore chips and the amino coupling kit were purchased from General Electric Healthcare Life Science (Piscataway, NJ). E*scherichia coli* DH5α and BL21 (DE3) strains were from Stratagene (La Jolla, CA, USA).

### Recombinant Expression and Purification of Superantigens

Streptococcal SSA and staphylococcal SEI and SEG were produced and purified as previously described (35, 65). Briefly, proteins cloned in pET-26b were expressed in *E. coli* BL21 and purified by Ni-NTA affinity chromatography, followed by S-200 molecular exclusion. Proteins were treated with agarose-polymyxin B (Sigma Aldrich, St. Louis, MO) to remove LPS traces. SEM and SEO were cloned and produced following the same strategy described earlier using strain 41,399.

### HLA-DR1 Expression and Purification

HLA-DR1 was produced by *in vitro* folding from bacterial inclusion bodies as described elsewhere (69). Briefly, plasmids encoding the HLA-DR1 α chain (DRA^*^0101) and β chain (DRB1^*^0101) were transformed separately into *E. coli* BL21 (DE3) cells (Stratagene). Bacteria were grown at 37°C to an absorbance of 0.6–0.7 at 600 nm, and then 1 mM IPTG was added. Inclusion bodies were extensively washed and the subunits purified under denaturing and reducing conditions using an HQ50 anion exchange column (PerSeptive Biosystems). Yields of DR1 α and β subunits were 16 and 20 mg per liter of culture medium, respectively. Purified subunits were diluted dropwise with constant stirring to a final concentration of 50 μg/ml into a folding solution of 20 mM Tris-HCl (pH 8.5), 25% (w/v) glycerol, 0.5 mM EDTA, 3 mM reduced glutathione, and 0.3 mM oxidized glutathione and kept at 4°C for 2 days in the presence of 1 μM HA 306–318 peptide (PKYVKQNTLKLAT). Recombinant HA/HLA-DR1 was purified from the folding mixture using a Mono Q anion exchange column (Amersham Biosciences) equilibrated with 20 mM Tris-HCl (pH 8.0) and developed with a linear NaCl gradient. The protein eluted as a single peak at 0.15 M NaCl.

### Purity of Recombinant Proteins and LPS Determination

**Figure 1:**
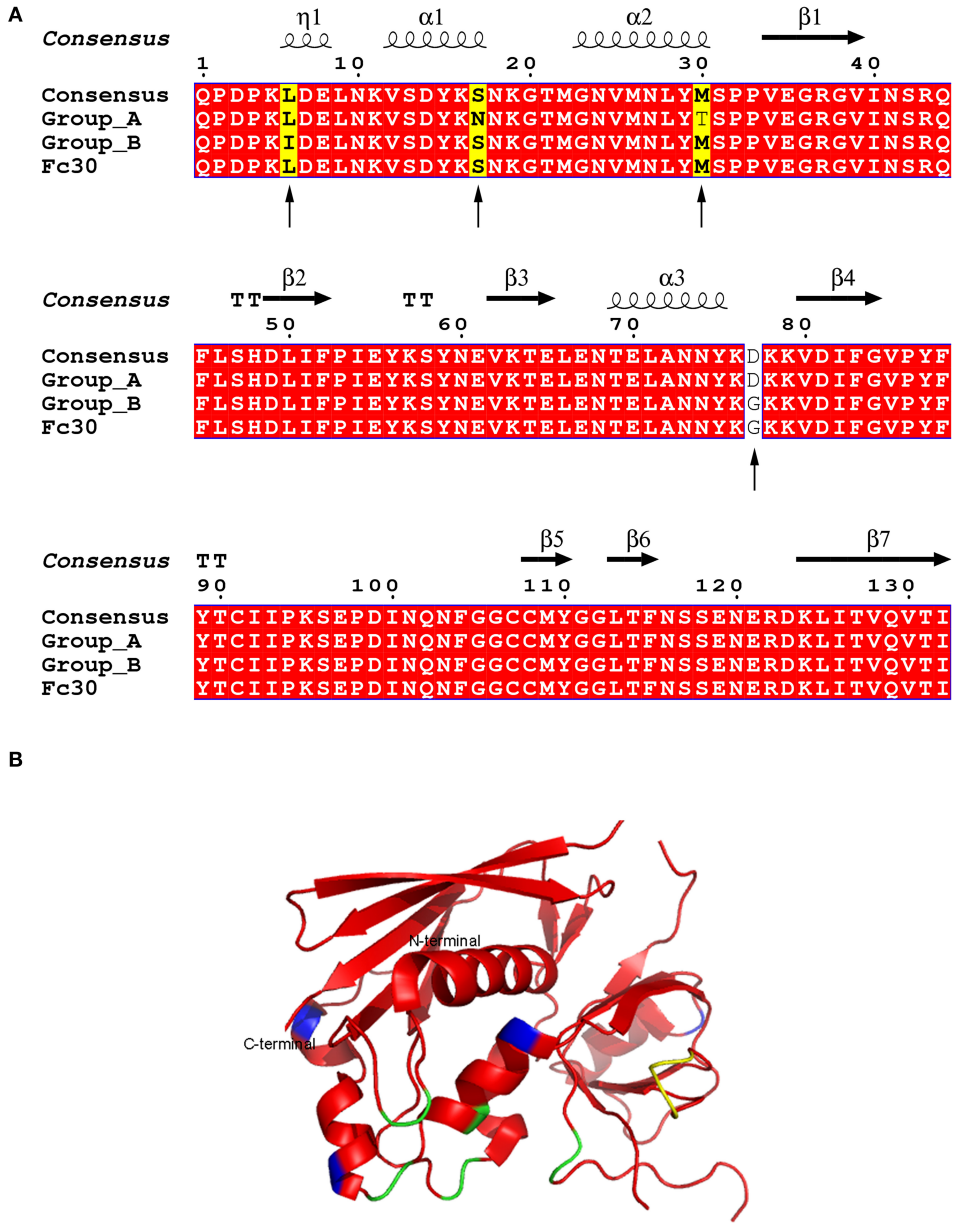
Amino acid alignment and structure of SEG variants. **(A)** SEG sequence labeled as consensus has been reported in four strains (N315, Q8RR77, Mu50, and FRI572) (18, 64, 71). Group A includes strains 40900, 41026, 41226, and 41399. Group B includes strains 41395, 41668, and 41664. Fc30 is used as reference for pediatric isolates as reported by (45). Identical residues are shown in *red*. *Arrows* show sequence sites containing mutations. Sites where a unique sequence is mutated are shown in *yellow*, and mutated amino acids in two sequences are shown in *white*. **(B)** Overall structure of SEG (pdb 1XXG). Mutations are colored in *blue*, MHC-II binding site in *yellow*, and TCR binding site in *green*.

For every recombinant protein used in this work, purity was evaluated by SDS-Page (egc SAgs in SP Figure 1). In addition, the amount of remaining endotoxin was evaluated by the Pierce LAL chromogenic endotoxin quantitation Kit (Thermo Scientific) following the manufacturers' instructions. For all recombinant proteins, it was verified that <0.1 EU/ml of endotoxin was present.

### Cell Culture

The human monocytic leukemia cell line THP-1 was obtained from the American Type Culture Collection (Manassas, VA) and cultured in complete medium [RPMI-1640 (Thermo Fisher Scientific), supplemented with 10% fetal bovine serum (FBS) (Internegocios S.A., B.A., Argentina), 2 mM glutamine, 1 mM pyruvate, 100 U/ml penicillin, and 100 μg/ml streptomycin].

When appropriate, and before each assay, cells (7.5 × 10^5^ to 10 × 10^5^ cells/ml) were treated for 72 h with phorbol 12-myristate 13-acetate (PMA, Sigma Aldrich, Saint Louis, MO) (40 ng/ml) or left untreated. Before each assay, cells were counted after trypan blue staining in a Neubauer chamber; only samples with cell viability over 98% were used.

### Cell Proliferation Assays

Cell inhibition assays were assessed in THP-1 cells, cultured in the presence of SAgs (from 0.001 to 10 μg/ml) or medium (control) for 24–72 h. For 48 h of treatment, cells were incubated for 30 h at 37°C in 5% (v/v) CO_2_. After that, 1 mCi of [^3^H] thymidine per well was added, and cells were incubated for additional 18 h and then harvested on glass fiber filters. The incorporation of radioactivity was measured using a liquid scintillation analyzer Tri-Carb 2810 TR (Perkin Elmer). To evaluate the specificity of our results and putative targets involved, inhibition assays were repeated with the same concentrations of SAgs, pre-incubated or not with specific F(ab)'_2_ fragments (10 μg/ml) and DR1 recombinant protein in equimolar concentration with the toxins assayed for 1 h at 37°C, and then incubated with cells for 48 h at 0.1 × 10^6^ cell/well.

### Immunofluorescence Assays

THP-1 cells (5 × 10^5^ cells/well) were incubated for 48 h with 10 μg/ml of each SAg or left untreated (control 1). Cells were fixed with PFA 2% (12 min at room temperature) and washed with PBS, and then the pellet was incubated for 30 min with 0.25% Triton X-100 for permeabilization. Blocking was performed by incubating 20 min at RT with 10% BSA-PBS. Primary antibodies, mouse sera anti Sags, or normal mouse sera (control 2) (dilution 1/300) were incubated for 1.5 h, then washed, and incubated with goat anti-mouse IgG conjugated with Alexa-488 (Biolegend) (dilution 1/1,000) for 1 h at RT. After final washes, the pellet was resuspended in DAPI (Sigma, St Louis, MO) (1 μg/ml) for 30 min. Samples were centrifuged, and a drop was observed under a fluorescent microscope (Olympus BX51TRF).

### Cell Death Assays

Cell viability was measured using the fluorescein diacetate (FDA) and propidium iodide (PI) assay, both purchased from Sigma (St. Louis, MO). FDA stain was used as a control of viability. Control and SAg-treated (10 μg/ml) cells were collected and washed twice in PBS before re-suspension in 1.4 μM of FDA and then in 1 μg/ml of PI. The percentage of dead cells up-taking PI was measured by flow cytometry (Partec, Germany) and analyzed using Flowing Software (Cell Imaging Core, Turku Centre for Biotechnology).

### Evaluation of Apoptosis by Annexin V

For assessment of apoptotic death, the PE-Annexin V Apoptosis Detection Kit I (BD Biosciences) was employed. Briefly, 5 × 10^5^ THP-1 cells were seeded in a 24-flat bottom plate and incubated with SAgs (10 μg/ml) or medium as a control for 48 h. Afterwards, cells were washed and resuspended in staining buffer. After 15 min of incubation with Annexin V-PE and 7-AAD, samples were injected into the flow cytometer, and results were analyzed using Flowing Software.

### Dual Acridine Orange/Ethidium Bromide Fluorescent Staining

To confirm the cell death rates using the PE-Annexin V Apoptosis Detection Kit I, we assessed the dual acridine orange/ethidium bromide (AO/EB) fluorescent staining, visualized under a fluorescent microscope (Olympus BX51TRF). THP-1 cells were treated with SAgs (10 μg/ml) for 48 h. The pelleted cell suspension (250 μl) was stained with 10 μl of the dye mixture [10 μM acridine orange and 10 μM ethidium bromide (Sigma)], which was prepared in PBS. Cells cultured with medium were used as a control. For THP-1/PMA-treated cells, the samples were evaluated attached to the cover glasses at the same time that the supernatants were evaluated. The morphology of 200 cells per sample was examined by fluorescent microscopy within 20 min.

### LDH Assay

Supernatants of THP-1 cells treated with SAgs for 48 h or left untreated were evaluated using the optimized UV method (DGKC) for lactate dehydrogenase (LDH): LDH-P (Wiener Lab.). Samples were evaluated at 340 nm and 37°C according to the manufacturer's indications.

### Cytokine Determinations

Cytokines (CKs) IL-6, IL-12, IFN-ɤ, TNF-α, IL-17A, and IL-10 were measured by ELISA from the supernatants (SN) of THP-1 cells incubated with different concentrations of SAgs (R&D Systems, Oxon, UK). Each experiment was repeated at least three times.

### Phenotypic Assay

THP-1 cells (10^6^/ml) were incubated with 1 μg/ml of SEG, SEI, or SSA for 24 h at 37°C in complete medium. CD14, CD40, and CD86 expression was evaluated by flow cytometry (FACS) and compared to basal expression in THP-1 cells incubated without SAgs (control). Briefly, we analyzed the THP-1 phenotype using FITC-labeled mAbs to CD40 and CD86 and PE-labeled antibodies to CD14 (eBioscience). Stained cells were analyzed by flow cytometry. At least 20,000 events were acquired for each sample, and data analysis was performed using the WinMDI software program. Results were expressed as percentage of positive cells.

### Phagocytosis Assays

THP-1 cells (10^6^/ml) were cultivated with 10^8^ UFC/ml of heat-dead *S. aureus* labeled with FITC in the presence of different SAgs (1 μg/ml) or alone. All the assays were done in complete medium. After 1–3 h at 37°C, CO_2_ 5% incubation, cells were washed and resuspended in PBS-Trypan Blue to analyze the phagocytosis of FITC-bacteria by flow cytometry. Results were expressed as percentage of positive cells.

### Surface Plasmon Resonance Assay

SPR analysis was performed using a BIAcore T100 instrument (GE Healthcare). Gp130 protein was immobilized (2200 RU) on a CM5 chip (GE Healthcare) surface by amine coupling according to the manufacturer's instructions. Soluble SEG, SEI, SEM, and SEO were diluted in PBS, pH 7.4, with or without EDTA running buffer, and injected over chip surfaces at a flow rate of 30 μl min^−1^ for 60 s at 25 °C. Data were analyzed with the BIA evaluation software (GE Healthcare).

### Statistical Analysis

Results were tested statistically using one-way and two-way ANOVA and Dunnett's, Bonferroni's, or Tukey's multiple comparison tests where appropriate, using commercially available software (Graph-Pad Prism, GraphPad Software, San Diego, CA, USA). Results were determined to be statistically significant when a *p* < 0.05 was obtained. Images were edited using Adobe Photoshop CC software (Adobe, CA, USA).

## Results

### SAgs of the *egc* Operon Are the Most Prevalent SAg Genes in the Evaluated Strains

To evaluate the prevalence of classical and non-classical staphylococcal *sags* in *S. aureus* isolated from adult patients of our community, we analyzed 13 strains from ambulatory patients who attended the emergency service of a public hospital in Buenos Aires (SP Table 2). All strains were microbiologically confirmed as *S. aureus* by coagulase reaction and production of clumping factor.

We assessed the presence of 15 different *sags* in the bacterial genomic DNA by PCR (Table 1). Classical *sags* were found in 60% of the isolates, with *sec3* showing the highest frequency. All strains were negative for *sea* and *see*. Within the non-classical *sag* genes, *ser* and *seh* were not detected in any of the analyzed strains when genomic DNA was evaluated.

**Table 1:** Distribution of *SAgs* genes in clinically isolated *Staphylococcus aureus* strains.

**Gene**																
**Strain**	***sea***	***seb***	***sec***	***sed***	***see***	***seh***	***sej***	***ser***	***tsst-1***	***seg***	***sei***	***sei 2***	***sem***	***sen***	***seo***	***seu***
40900	–	–	**+**	–	–	–	**+**	–	–	**+**	**+**	–	**+**	**+**	**+**	**+**
41026	–	–	**+**	–	–	–	–	–	–	**+**	**+**	–	**+**	**+**	**+**	**+**
41192	–	–	–	–	–	–	–	–	–	–	–	–	–	**+**	–	–
41226	–	–	**+**	–	–	–	–	–	**+**	**+**	**+**	–	**+**	**+**	**+**	**+**
41395	–	–	–	–	–	–	–	–	–	**+**	–	**+**	**+**	**+**	**+**	**+**
41399	–	–	**+**	–	–	–	–	–	–	**+**	**+**	–	**+**	**+**	**+**	**+**
41524	–	–	–	–	–	–	–	–	–	–	–	–	–	**+**	–	**+**
41598	–	–	**+**	–	–	–	–	–	–	–	–	–	–	–	–	–
41627	–	**+**	–	**+**	–	–	–	–	–	–	–	–	–	**+**	–	–
41668	–	–	**+**	–	–	–	**+**	–	–	**+**	–	–	**+**	**+**	**+**	**+**
41674	–	**+**	**+**	–	–	–	–	–	–	**+**	**+**	–	**+**	**+**	–	**+**
41759	–	–	–	–	–	–	–	–	–	–	–	–	–	**+**	–	–
41762	–	–	–	–	–	–	–	–	–	–	–	–	–	**+**	–	–
**%**	0	15	54	8	0	0	15	0	8	54	38	8	54	92	46	62

The presence of the *egc* operon was detected in 54% of the strains. *sen* [previously named as *sek* (54)] a gene that can or cannot be present in the *egc* operon, showed the highest prevalence (92%), followed by *seu* (62%). A non-significant relationship was found between the severity of the infection or the strain antibiotic resistance and the presence of *sags*.

These results are in concordance with several reports that claimed that 80% of all *S. aureus* strains hold, on average, five or six SAg genes, among which the *egc* SAgs are the most prevalent today (50–55).

The *egc* operon was described for the first time by Jarraud et al. (54). At that time, three forms of the *egc* operon were described: *egc1* (harboring *seg, sei, sem, sen, seo*, and *ent1* and *ent2*), *egc2* (harboring *seu* instead of *ent1* and *ent2*), and *egc3* (containing *sei, seu, sen* and *seg* variants). Blaiotta et al. (70) described *egc* forms 1–7 based on the length of the egc DNA fragment and its restriction enzyme assay patterns. Here, we described a non-reported form of the *egc* operon lacking the *seo* gene, which we denominated *egc8*, in accordance with previous works. Additionally, we found that *sen* was observed no matter if any form of the cluster was present or not (92% prevalence compared with 54% prevalence of the *egc* operon). Furthermore, we found the presence of a reported variant of *sei, sei2* (49, 52).

*seg* was found in seven isolated strains displaying slight DNA variability with mutations in residues 6, 17, 30, and 77 (Figure 1A) when compared with the consensus sequence (18, 64, 71). These mutations allowed us to group them as A, which includes isolated strains 40,900, 41,026, 41,226, and 41,399 (accession numbers MK947360, MK947361, MK947362, and MK947364) and B, which includes strains 41,395, 41,668, and 41,674 (accession numbers MK947363, MK947365, and MK947366) (Figure 1A). The mutations in position 6 (Ile instead of Leu) and 30 (Thr instead of Met) are conservative since the biochemical nature of the amino acids did not change with respect to the consensus sequences. The mutation in residue 77 is identical to the one observed in pediatric isolates (Fc30) (35). All these residues are highly solvent-exposed, and neither the MHC-II binding site nor the TCR binding surfaces were affected by the mutations, according to the elucidated structure (Figure 1B).

On the other hand, *sei* showed the same sequence as the consensus in the two variants of the gene previously described.

### SAgs Inhibit Monocyte Proliferation

**Figure 2:**
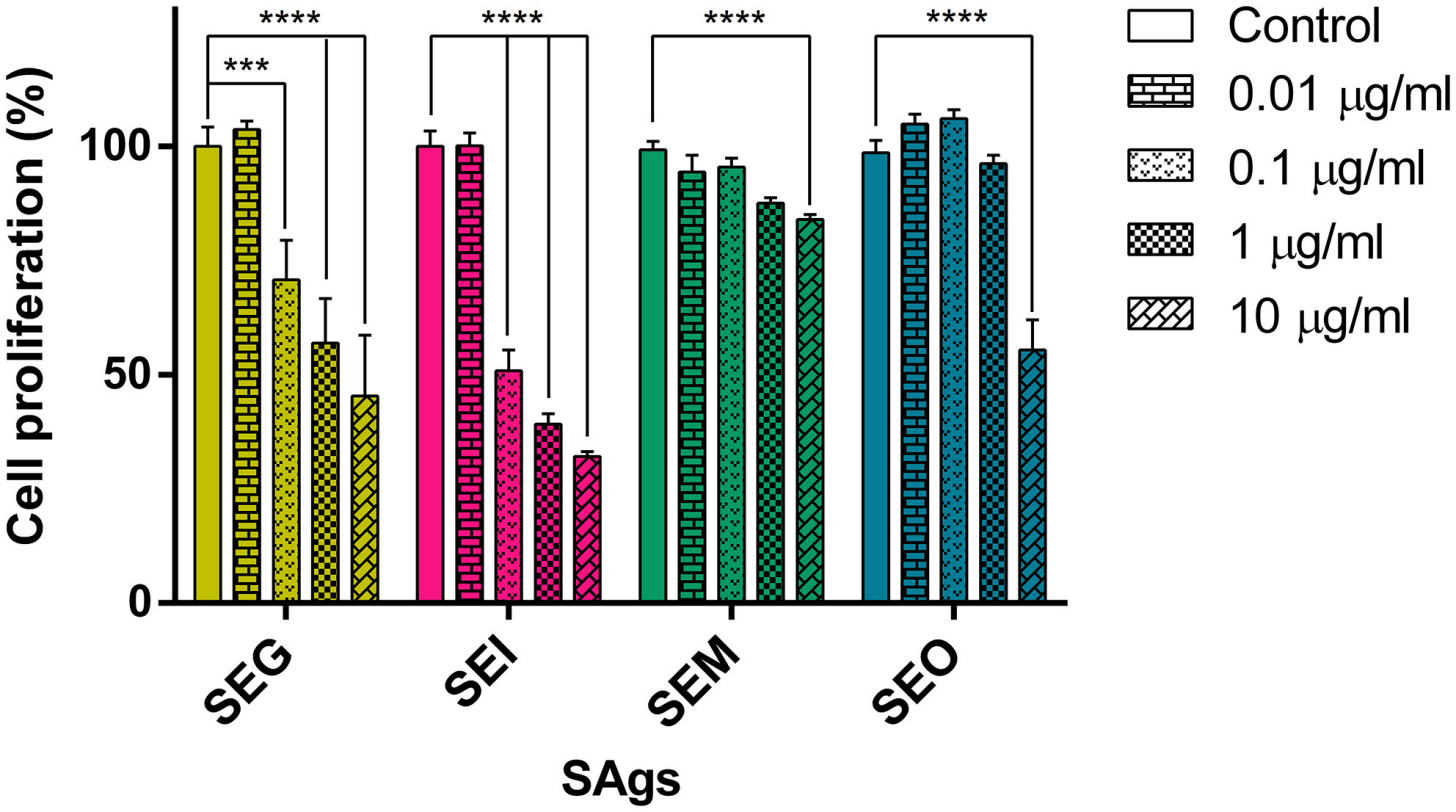
SAgs inhibit proliferation of THP-1 cells. THP-1 cells were incubated with Sags, and proliferation by [^3^H]-thymidine was evaluated at 48 h. Results are expressed as the percentage of proliferation related to untreated conditions. Data are expressed as the mean ± SEM of at least three independent experiments. *Asterisks* represent statistical significance with respect to untreated cells: ***p < 0.001, ****p < 0.0001.

Four SAgs of the *egc* operon were cloned in pET-26b, successfully expressed in transformed *E. coli* BL21 and purified (SP Figure 1). In order to study the effect of SAgs on innate immune cells and in the absence of T cells, a human monocyte cell line (THP-1) was cultured for 48 h in the presence of the four recombinant SAgs, and proliferation by [^3^H]-thymidine was evaluated. Since *sen* and *seu* are not always present with the *egc* operon, they were not included in this study. All SAgs assayed caused lower proliferation rates in relation to control (untreated cells), although different potencies in their effects were observed. Figure 2 shows a representative result for 48 h culture of SAg with 0.2 × 10^6^ cells/well where inhibition of proliferation was observed at as low as 0.1 μg/ml for SEG and SEI and at 10 μg/ml for SEM and SEO. Similar results were obtained for different cell densities (0.2–1.0 × 10^6^ cells/well).

### All SAgs Induce Monocyte Death

**Figure 3:**
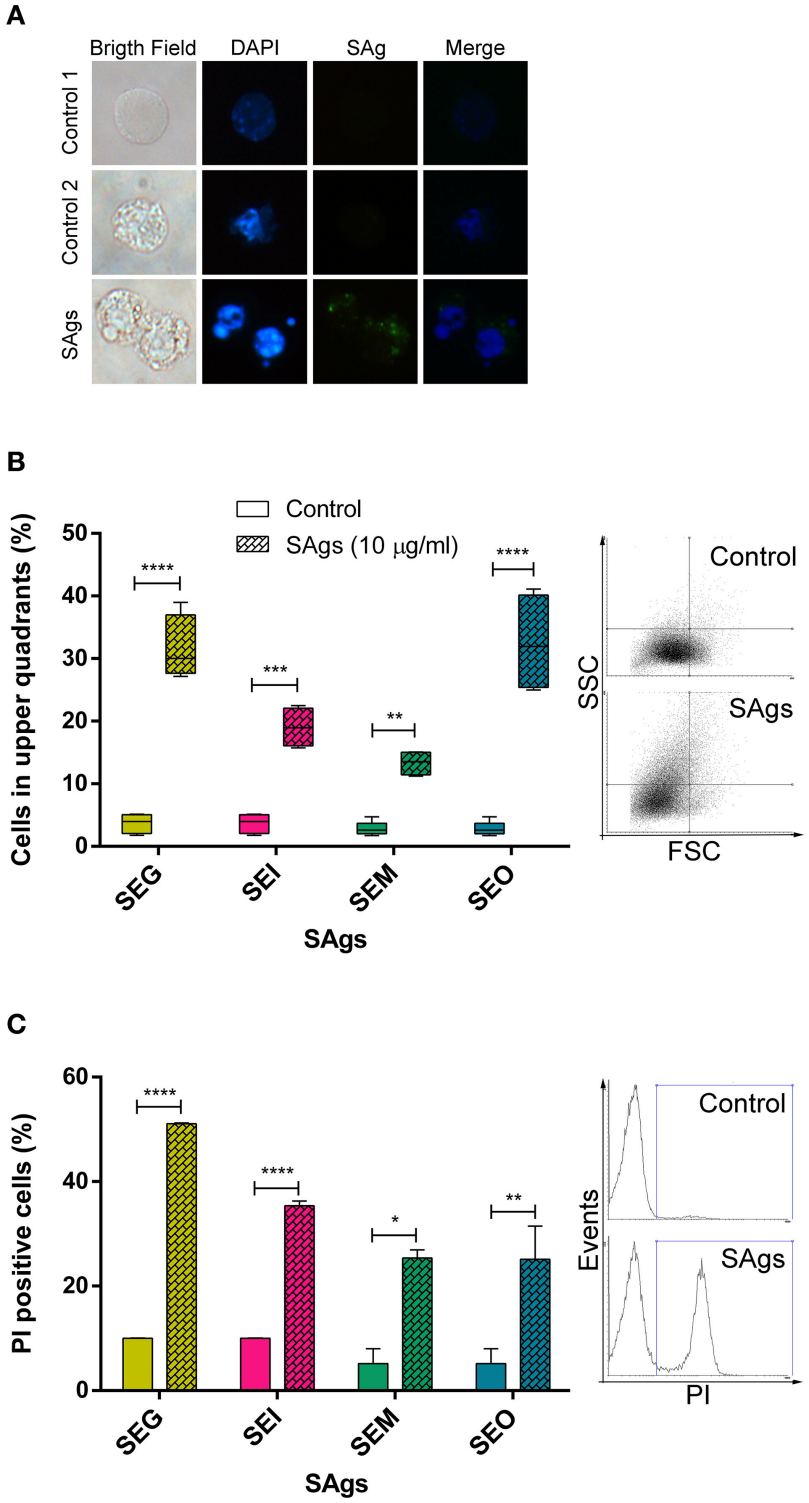
SAgs of the *egc* operon induced monocytic cell death. **(A)** After 48 h of incubation with SAgs, THP-1 cells were labeled with fluorescent antibodies and observed under the microscope; DNA is shown in *blue* and SAgs in *green*. In the first *column*, images in bright field are shown. The upper panel shows the control condition with untreated cells. In the middle and lower panels, monocytes treated with SAgs are shown. In the middle panel, control 2, cells were incubated with a normal mouse serum and then stained with fluorescent antibodies. In all cases, representative images are shown. **(B)** Cellular complexity was determined as an increment of cells in the upper quadrants of FSC vs. SCC dot plot. THP-1 cell complexity was assessed by flow cytometry after 48 h of incubation with SAgs. For this purpose, THP-1 cells were gated for singlets, and then the monocytic population was evaluated by FSC and SCC. Values are expressed as percentage of cells in the upper quadrants. Representative *dot plots* are shown on the right. **(C)** THP-1 cells were incubated with SAgs for 48 h and then stained with FDA/PI. In this case, after singlet cell and monocytic population selection, the percentage of PI^+^ cells was measured for every SAg treatment and its basal. Values are expressed as the percentage of PI^+^ cells. Representative *histograms* are shown on the right. Data are expressed as the mean ± SEM of at least three independent experiments. *Asterisks* represent statistical significance with respect to untreated cells: *p < 0.05, **p < 0.01, ***p < 0.001, ****p < 0.0001.

We further investigated if SAgs were only responsible for inhibition of proliferation or if they were also causing cell death. At first, we observed DNA conformation and SAgs presence in THP-1 cells by immunofluorescence assay. THP-1 cells charged with SAgs in their cytoplasm presented nuclear fragmentation, indicating cell death. Untreated cells were used as a biological control (control 1) and SAgs-treated cells incubated with normal murine sera were used to confirm assay specificity (control 2) (Figure 3A). In addition, an increment in cellular complexity was observed by flow cytometry (Figure 3B). This phenomenon was observed for all *egc* SAgs, suggesting intracellular events that could be related to the cell death described above. Moreover, when THP-1 cells were cultured in the presence of 10 μg/ml of SAgs for 48 h, all toxins showed an increased percentage of PI-positive THP-1 cells, indicating cell death induction (Figure 3C).

We observed that SEG was the toxin inducing more changes in cellular complexity and an increase in cell death by PI. Moreover, SEM induced the lowest effects in both evaluated situations.

**Figure 4:**
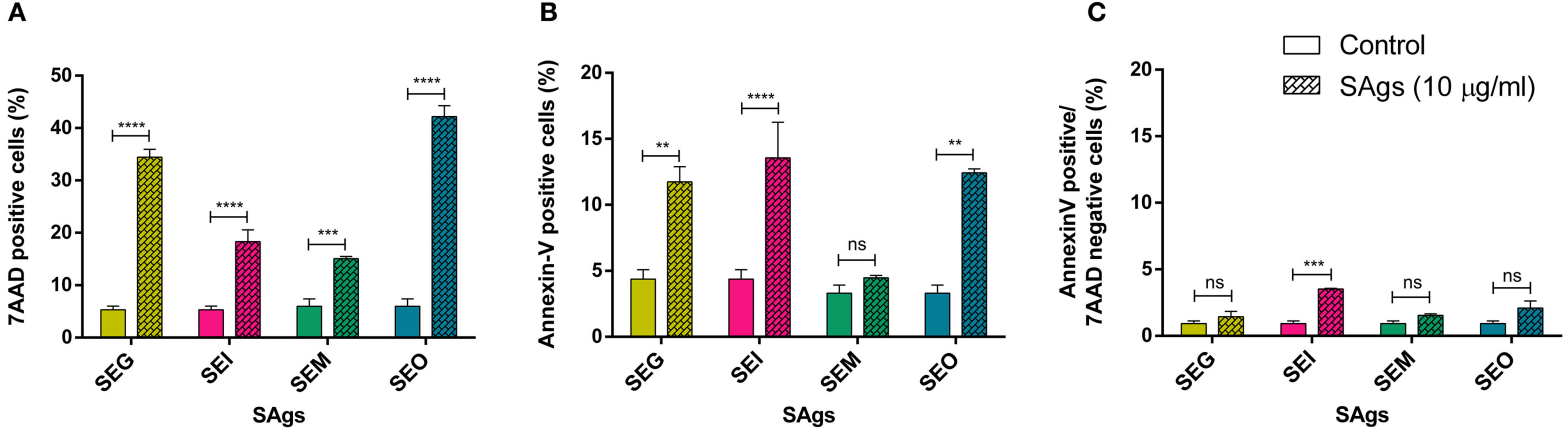
SAgs induced cell death in THP-1 cells by different mechanisms. After 48 h of incubation with SAgs, THP-1 cells were incubated with Annexin V-PE and 7AAD, and the proportion of positive cells was measured before the singlet and monocytic population gating strategy. Values are expressed as the percentages of 7AAD positive cells **(A)**, Annexin V positive cells **(B)**, and Annexin V-positive/7AAD-negative cells **(C)**. Data are expressed as the mean ± SEM of at least three independent experiments. *Asterisks* represent statistical significance with respect to untreated cells: **p < 0.01, ***p < 0.001, ****p < 0.0001, ns, non-significant difference.

PI-positive cells are usually considered as necrotic cells, but primary necrotic and post-apoptotic secondary necrotic cells could not be discriminated by this method (72). To clarify this point, we performed Annexin V/7AAD and dual acridine orange/ethidium bromide fluorescent staining. When THP-1 cells were cultured in the presence of 10 μg/ml of SAgs for 48 h, all toxins increased the percentage of 7AAD positive THP-1 cells (Figure 4A). All SAgs, but SEM, increased the proportion of cells labeled with Annexin-V (Figure 4B); however, only SEI interestingly showed a low percentage of early apoptosis (Figure 4C).

**Figure 5:**
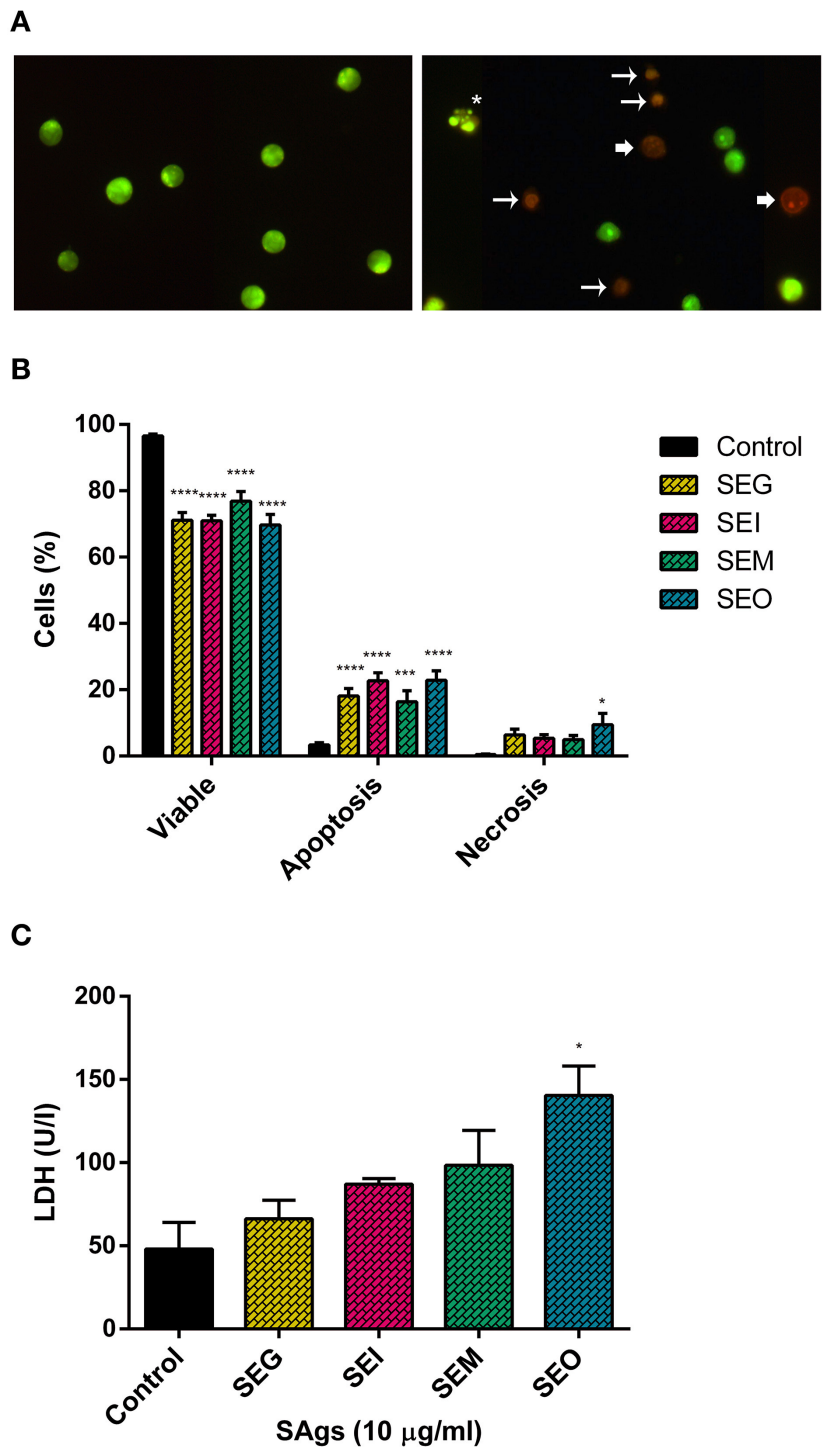
SAgs induced cell death in THP-1 cells by apoptosis and some necrosis. Acridine orange-ethidium bromide staining of monocytic cells (THP-1) under different treatment conditions (representative images). **(A)** Control THP-1 cells (left panel) and SAgs treatment (right panel) are shown. *Asterisks* indicate early apoptosis cells, *regular arrows* show late apoptosis cells, and *wide arrows* show necrosis. **(B)** Viability and type of death percentage of THP-1 cells treated with SAgs or untreated (control). The result is based on the analysis of live and apoptotic/necrotic cells following acridine orange-ethidium bromide (AO/EtBr) staining. **(C)** Analysis of LDH assay of THP-1 cells treated with SAgs or left untreated. *p < 0.05, ***p < 0.001, ****p < 0.0001.

Furthermore, THP-1 cells treated with SAgs followed by AO/EtBr staining showed a viability reduction of the monocytic cells treated with the four SAgs (Figures 5A,B). SEI and SEO showed the highest increment in apoptotic cell death, compared with SEG and SEM. In contrast, although all SAgs showed an increase of cells undergoing necrosis; only SEO increase was significantly higher compared to control. In correspondence with that, only SEO increased the level of LDH measured in THP-1 supernatants (Figure 5C).

Thus, we corroborated the induction of cell death generated by these toxins, causing the depletion of effector cells from the innate immune response by multiple mechanisms. While the four toxins induced late apoptosis at 48 h, we observed induction of early apoptosis by SEI and necrosis increment by SEO.

### SAgs Induce a Pro-inflammatory Cytokine Profile in Monocytes

**Figure 6:**
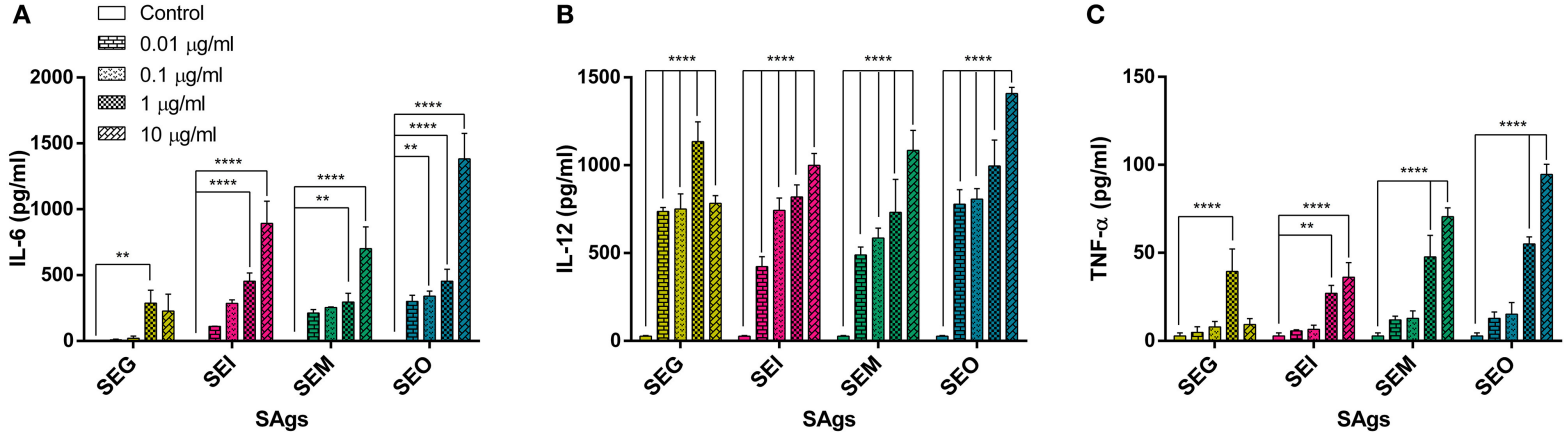
All SAgs induced production of pro-inflammatory cytokines by THP-1 cells. Cytokines were measured in supernatants of treated cells; values are expressed in pg/ml. IL-6 **(A)**, IL-12 **(B)**, and TNF-α **(C)** production is shown. No production of IL-10, IL-17, or IFN-γ was detected in any case. Data are expressed as the mean ± SEM of at least three independent experiments. *Asterisks* represent statistical significance with respect to untreated cells: **p < 0.01, ****p < 0.0001.

When supernatants of THP-1 cells cultured in the presence of SAgs for 48 h were analyzed, we observed a significantly higher production of cytokines IL-6, IL-12, and TNF-α (Figure 6). These interleukins propitiate a highly inflammatory environment, proper to eradicate an intracellular bacterium, while *S. aureus* spends most of the time as an extracellular pathogen. On the contrary, no trace of IL-17A, IFN-γ, or IL-10 was detected (results not shown). Evidently, there is cell activation at the same time that death process starts to occur.

Interestingly, SEG was the weakest to induce IL-6 production by THP-1 cells and in all cases showed a decline in cytokine levels at the highest concentrations (Figure 6).

### SAgs Induce Cell Death in Monocytes Differentiated Into Macrophages

Since macrophages are the major phagocytic cells, THP-1 cells were differentiated into them by treatment with PMA and then incubated with SAgs to evaluate their effects.

**Figure 7:**
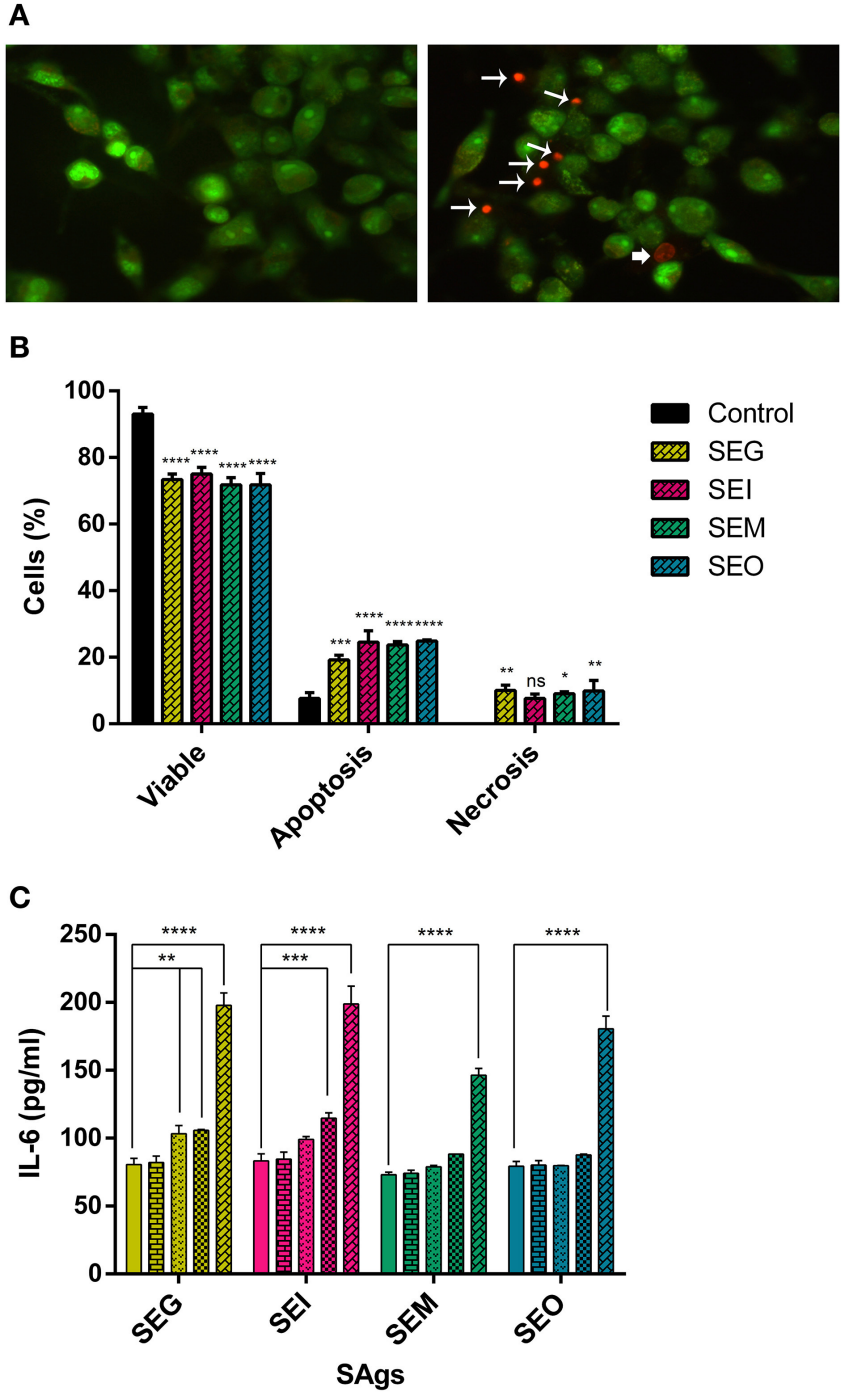
PMA THP-1 differentiated cells are more sensitive than THP-1 cells to the SAg effect. Acridine orange-ethidium bromide staining of PMA-treated THP-1 cells under different treatment conditions (representative images). **(A)** Control THP-1/PMA cells (left panel) and SAgs treatment (right panel) are shown. *Asterisks* indicate early apoptosis cells, *regular arrows* show late apoptosis cells, and *wide arrows* show necrosis. **(B)** Viability and type of death percentage of THP-1/PMA cells treated with SAgs or untreated (control). The result is based on the analysis of live and apoptotic/necrotic cells following acridine orange-ethidium bromide (AO/EtBr) staining. **(C)** Cytokines were measured in supernatants by ELISA; production of IL-6 is shown. Values are expressed in pg/ml. Data are expressed as the mean ± SEM of at least three independent experiments. *Asterisks* represent statistical significance with respect to untreated cells: *p < 0.05, **p < 0.01, ***p < 0.001, ****p < 0.0001.

To evaluate cell death, AO/EtBr assay was performed (Figures 7A,B). THP-1 cells treated with PMA and incubated with SAgs for 48 h followed by AO/EtBr staining showed a reduction on the macrophage-like cell viability. Although the effect on viability is less marked than with THP-1 cells, all SAgs augmented the percentage of death by apoptosis, with SEG being the one that induced the lowest increment. In contrast, even though all SAgs showed an increase in the percentage of cells undergoing necrosis, that increment was significantly higher than the control for SEG, SEM, and SEO, but not for SEI.

All SAgs of the *egc* operon induced macrophage production of IL-6 in a concentration-dependent manner, but SEG and SEI increased the production of this cytokine at lower doses than SEM and SEO (Figure 7C). Production of IL-12, IFN-γ, or IL-10 was not detected in any case (results not shown).

### Monocyte Damage Is Mediated by SAgs Interaction With DR1 HLA Class II Molecules

**Figure 8:**
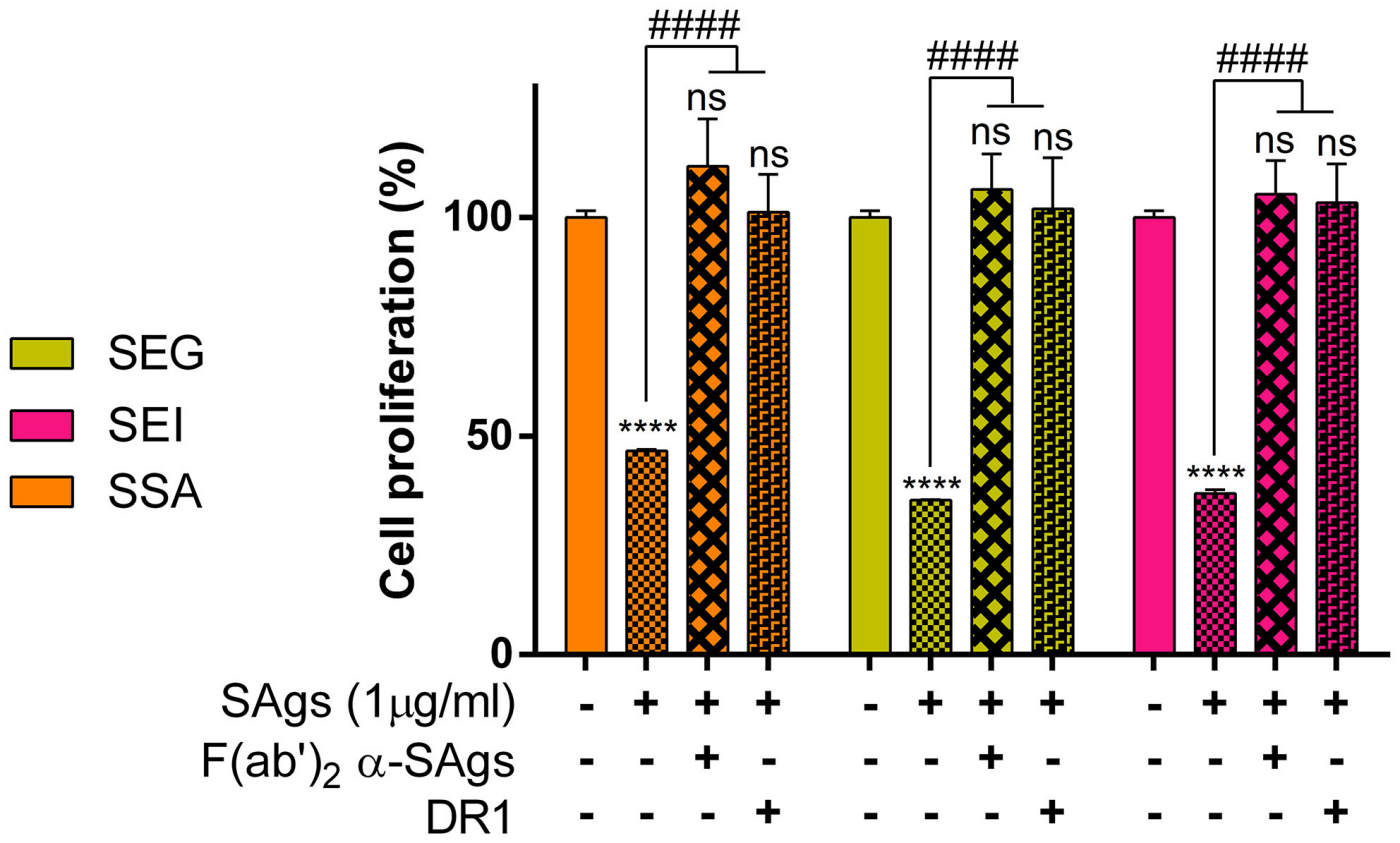
Inhibition of cell proliferation is prevented when SAgs are preincubated with HLA-DR1 or F(ab)'_2_. THP-1 cells were incubated with SAgs alone or with SAgs preincubated with DR1 or F(ab)'_2_ α-Sags, and proliferation was evaluated by [^3^H]-thymidine at 48 h. Results are expressed as the percentage of proliferation related to untreated condition (basal) for each SAg. In this experiment and the following, only the most studied *egc* SAgs, SEG, and SEI and streptococcal SSA were assessed. Data are expressed as the mean ± SEM of at least three independent experiments. *Asterisks* represent statistical significance with respect to the untreated cells in each treatment: ****p < 0.0001, ns, non-significant difference. *Hashes* represent statistical significance with respect to cells treated only with SAgs in each treatment: ^####^*p* < 0.0001.

With the aim of verifying whether the SAg effect on monocytes could be extended to streptococcal superantigen A (SSA), we pre-incubated THP-1 cells with SSA and found 60% inhibition of cell proliferation, similar to that obtained with staphylococcal SAgs (Figure 8). SSA inhibition of proliferation, as well as SEG and SEI inhibition, was abrogated by incubation with F(ab)'_2_ portion of specific polyclonal antibodies for each SAg (Figure 8).

Since the interaction of SEG and SEI with DR1-HLA, an isoform of the HLA class II molecule expressed on the THP-1 cell surface, is well-documented (35, 45, 65), we analyzed if SAgs damage to monocytic cells could be attributed to the binding of DR1. In addition, we also analyzed if the SAg SSA from *S. pyogenes* displayed a similar effect on monocytic cells. We had previously observed that SSA caused inhibition of proliferation similar to the SAgs of the *egc* operon (results not shown). SAgs and recombinant DR1 were pre-incubated at equimolar concentration for 60 min at 37°C, and the effects of the complex on THP-1 cells were analyzed by proliferation assay. The results in Figure 8 show that the pre-incubation of SAgs with the DR1 molecule inhibits the SAg effects on monocytes when proliferation is measured. These results strongly suggest that the SAg binding site with DR1 is required for THP-1 cell damage mediated by these toxins.

### SAgs Induce the Upregulation of Different Cluster Differentiation Markers Through Molecules Other Than DR1

**Figure 9:**
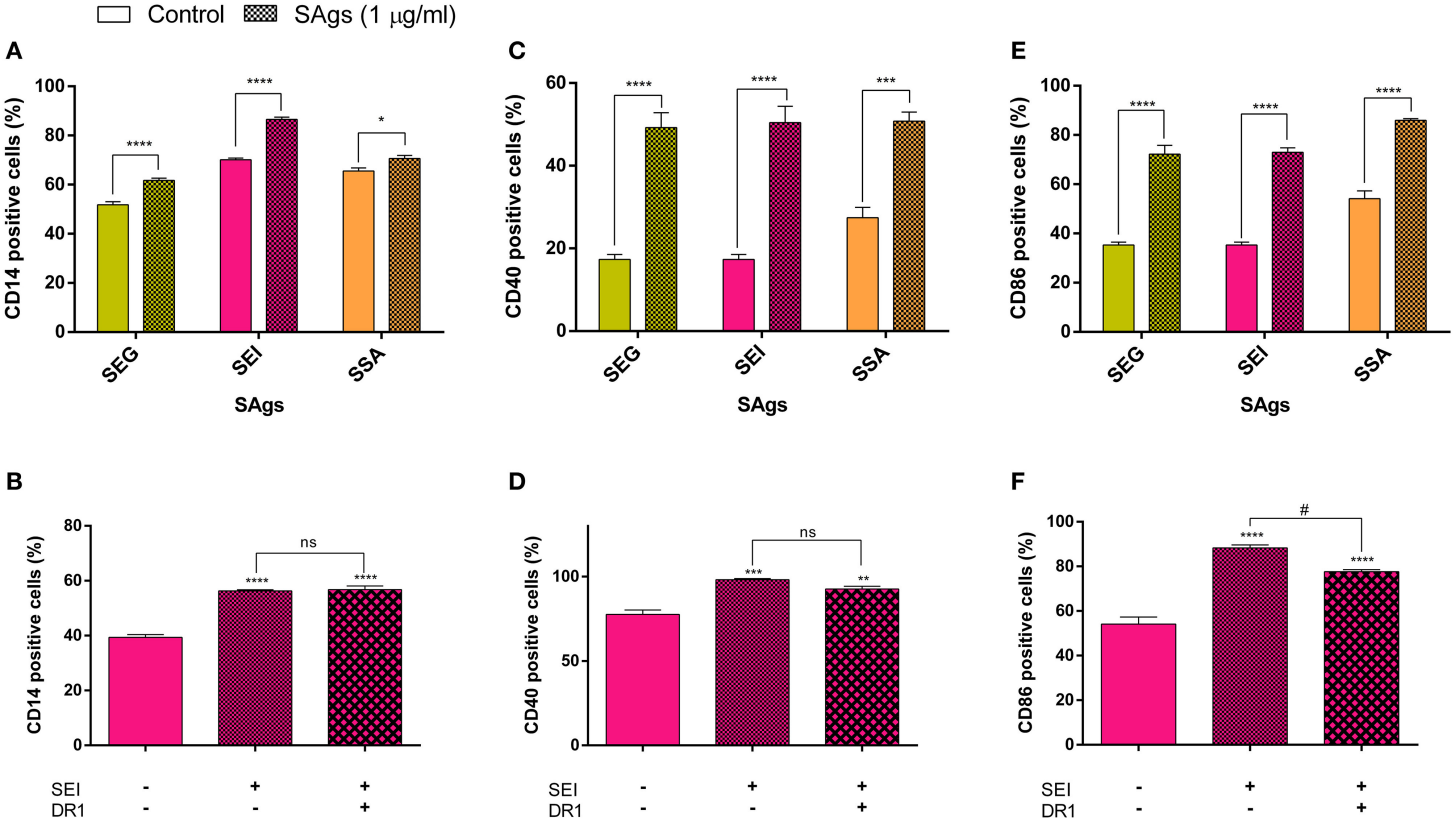
SAgs affect the expression of cluster differentiation (CD) markers. THP-1 monocytic cells were treated for 48 h with SAgs and then were incubated with antibodies to CD14 **(A)**, CD40 **(C)**, and CD86 **(E)**. Moreover, using SEI as a representative SAg, we performed the same assay with pre-incubation of SEI with DR1 in order to evaluate its effect on CD expression **(B,D,F)**. Specific interaction of SEI and tSEI was evaluated by SPR. Cytometry results were expressed as the percentage of positive cells for each marker. Data are expressed as the mean ± SEM of at least three independent experiments. *Asterisks* represent statistical significance with respect to the untreated cells in each treatment: *p < 0.05, **p < 0.01, ***p < 0.001, ****p < 0.0001, ns, non-significant difference. Hashes represent statistical significance with respect to treated cells with SAgs alone: ^#^*p* < 0.05, ns, non-significant difference.

All evaluated SAgs at 1 μg/ml had the capacity to induce the increase of CD14, CD40, and CD86 markers on THP-1 cells. CD14-positive cells were increased between 5 and 15% by SAgs with respect to control (untreated cells) (Figure 9A). CD14 ultimately activates NF-κB, and its overexpression shows a more mature stage of monocytes, which are shown slightly activated here.

CD40 signaling plays an important role in the proliferation and differentiation of B cells, and its upregulation implies monocytic activation. An increment of at least 100% of CD40-positive monocytes was observed after incubation with SAgs, with respect to control (Figure 9C).

In concordance, the co-stimulatory molecule CD86 is upregulated up to 50% by SAg incubation (Figure 9E). CD86 induces lymphocyte activation, thereby triggering cellular immune response, although it has also been implicated in immune regulation.

The upregulation of CD14 and CD40 did not disappear when SAgs were pre-incubated with DR1 before incubation with THP-1 cells (Figures 9B,D), suggesting that there are other possible molecular targets for SAgs on the surface of monocytes, which trigger the augmented expression of these molecules. For CD86, the upregulation caused by SAgs is partially prevented when pre-incubated with DR1 (Figure 9F), also indicating more than one target implicated in this molecule regulation.

### Phagocytosis Is Not Induced by Incubation With SAgs

When pre-incubated with SEG, SEI, or SSA, THP-1 cells did not change the ratio of phagocytosis of heat-killed bacteria (SP Figure 2). This result shows that, although an activated profile can be seen, the effector profile of these cells is not well-developed, as could be suggested by previous results.

### Gp130, a Glycoprotein Expressed on the Monocytic Cells, Binds Non-classical SAgs

**Figure 10:**
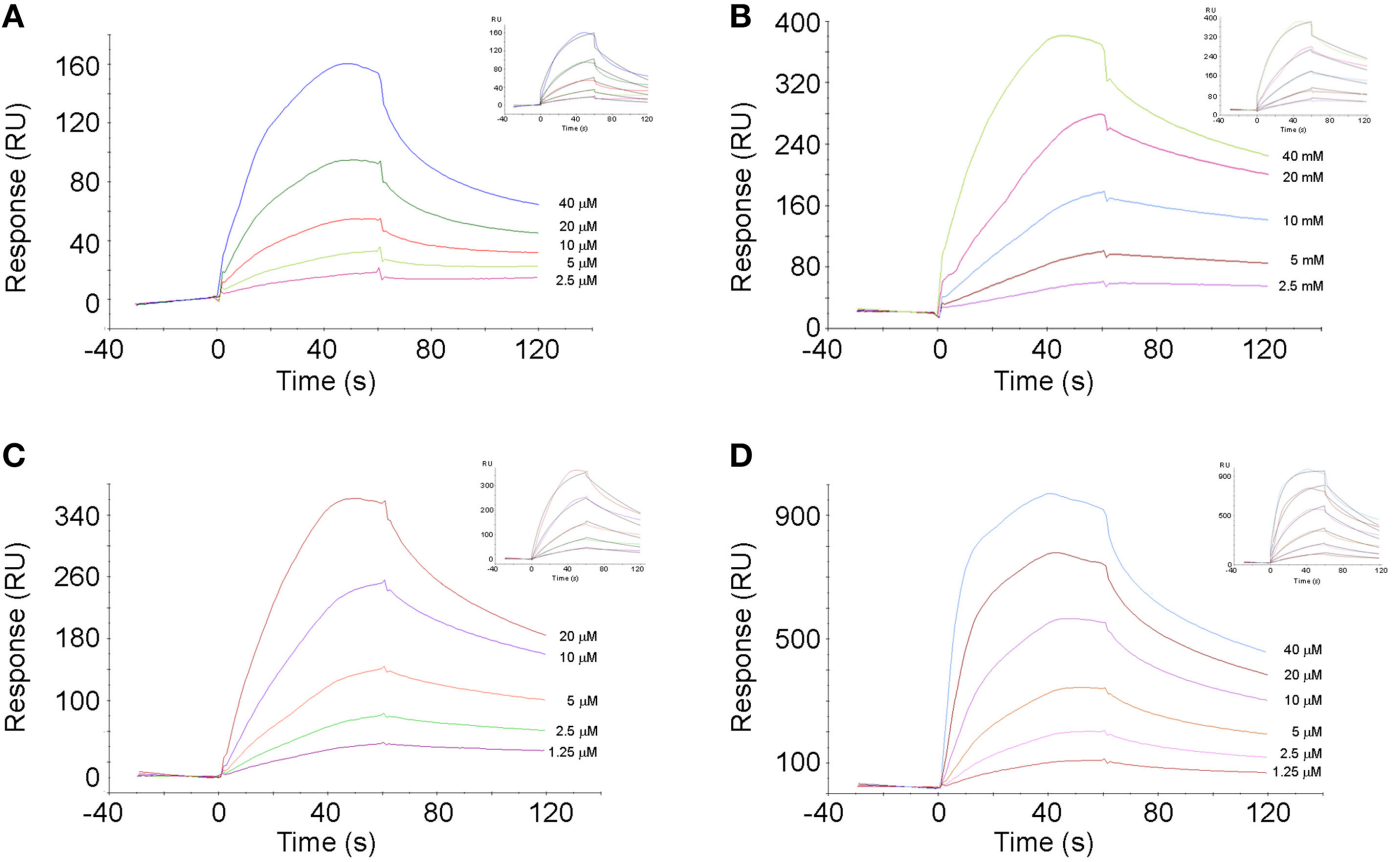
Specific interaction of SAgs with gp130. Sensorgrams of gp130–SAgs interaction are shown. Specific interaction between gp130 and SEG **(A)**, SEI **(B)**, SEM **(C)**, and SEO **(D)** are shown. The adjustments of experimental data to an interaction model 1:1 are shown as *insets*.

The molecule gp130, a glycoprotein expressed on monocyte cell surface (73), as described as a possible target of classical SAgs having an Asp227, a residue which is also involved in the binding to the β chain of the DR1 (48). Considering that SEI, a non-classical SAg, possesses an Asp in the homologous position (Asp209) (45), we analyzed SEI interaction with gp130 by SPR. One-fold dilutions of SEI (40–2.5 μM) were passed through gp130 immobilized on a CM5 chip surface displaying a specific interaction (Figure 10). The kinetic 1:1 interaction model displayed unambiguous results exhibiting a *K*_D_ of 1.7 × 10^−5^ M, with a very slow association rate (*k*_on_) of (8.3 ± 0.2) × 10^2^ (Ms)^−1^ and a moderate dissociation rate (*k*_off_) of (13.8 ± 0.3) × 10^−3^ s^−1^. Even though the calculations of the rates were univocal, the model did not display a good fit to the experimental values. When a second model, assuming a cooperative model or a 1:2 interaction was applied, a similar value of *K*_D_ was obtained. Considering that the interaction between DR1 and SEI is mediated by Zn, we also performed SPR in the presence of EDTA to sequester the cation (45), and the binding was not avoided (data not shown), suggesting that the SEI-gp130 interaction is independent of Zn.

Since the interaction between gp130 and SAg was only described for SEA and it was suggested that this interaction would depend on the presence of Asp227, we analyzed if the *egc* SAg SEG, which lacks Asp227 and binds to the DR1 α chain (35), also interacts with gp130. Surprisingly, SEG showed a higher affinity for gp130 than SEI, displaying a faster *k*_on_ and a slower *k*_off_ than SEI (Table 2, Figure 10). SEM and SEO also displayed a specific interaction with gp130 (Figure 10) with *K*_D_ values similar to that calculated for the SEG-gp130 interaction. The affinity between SEG, SEM, or SEO with gp130 did not show significant differences (Table 2).

**Table 2:** SAgs interaction with Gp130 by SPR.

	**gp130**		
**SAgs**	***K*_**on**_ (Ms)^**−1**^**	***K*_**off**_ (s)^**−1**^**	***K*_**D**_ (M)**
SEI	(8.3 ± 0.2) × 10^2^	(13.8 ± 0.3) × 10^−3^	1.7 × 10^−5^
SEG	(11.9 ± 0.1) × 10^2^	(6.2 ± 0.1) × 10^−3^	5.2 × 10^−6^
SEM	(19.9 ± 0.1) × 10^2^	(9.9 ± 0.4) × 10^−3^	5.0 × 10^−6^
SEO	(22.7 ± 0.6) × 10^2^	(12.6 ± 0.2) × 10^−3^	5.6 × 10^−6^

## Discussion

In the current study, we determined that the *egc* operon is frequently found in the 13 analyzed strains, in concordance with an extensive prospective study in Colombia (74). In contrast to this study, our results in the analyzed strains demonstrated that *sen* (previously *sek*) was more frequent than the operon itself. All the analyzed strains carrying the *egc* operon are also bearers of *seu*, increasing the virulence of the *S. aureus* strains (18, 49). This phenomenon could be explained in terms of the increment of vβ TCR isoforms interacting with different SAgs, which allows a major activation of the T cell population. Former publications described seven types of *egc* operon. Here we found a new form of the operon, named *egc 8*, which is characterized by the absence of the *seo* gene.

Although sed and *sej* can be encoded in the same plasmid (71), we found them separately in the genomic DNA of two different strains. These results are not surprising as many types of *sags* are encoded in mobile genomic elements which allow the integration into chromosomal DNA.

In general, staphylococcal *sag* genes are well-conserved and display minor allelic variations. However, we have previously described new allelic variants between *seg* and *sei* genes in Argentinean autochthonous strains (35). Pediatric isolates revealed mutations that were expressed as up to 10 changes in the SEI amino acid sequence and two in the SEG amino acid sequence. In this study, we observed that adult isolates displayed lower variability in the DNA sequence which resulted in non-variations detected for the *sei* nucleotide sequences compared with the consensus and four mutations distributed in pairs in SEG predictive mature proteins. These results could suggest that *sag* genes obtained from strains infecting adults and ambulatory patients displayed much lower variability than strains isolated from pediatric patients. The mutations detected in the SEG predictive mature protein altered residues located outside the MHC-II binding site or the TCR contact surface. These mutations appear as residues highly exposed to the solvent, located in mobile loops in the quaternary structure of the protein and very accessible to antibody recognition, suggesting a target to avoid the neutralization in future infections (9, 35, 75).

The SAg effects on T cells are very well-documented; however, the actions of these toxins on cells of the innate immunity at the early stages of infection when T cells are absent have not been deeply evaluated. Previously, our group described the interaction between SEG and mouse dendritic cells (mDCs) (59) using an *in vivo* and *in vitro* model. SAgs activate mDCs, increasing the phagocytosis process and inhibiting its maturation. In addition, we demonstrated that SAgs enter in the DC, avoiding the classical pathway of antigen degradation, and are finally exposed as intact molecules on the DC surface, a situation that would allow the interaction with MHC-II and TCR when the DCs encounter the T cells in the lymph nodes (59). However, as far as we know, SAgs impact on other isolated innate immune cells, such as human monocytes and macrophages, had not been studied yet.

Considering that the *egc* operon is the most frequent SAg cluster detected in autochthonous strains of *S. aureus*, we evaluated the activity of its SAgs over monocytes and macrophages using the THP-1 cell line as a model of human monocytes. A previous work reported that the interaction between human monocytes enriched from PBMCs and the staphylococcal enterotoxin B (SEB) induced monocyte apoptosis and secretion of IFN-γ (56). However, the presence of T cells, source of IFN-γ (76), in those preparations cannot be discarded. In the present work, SEG, SEI, SEM, and SEO inhibit monocyte basal proliferation in a dose-dependent manner, promoting cell death mostly by an apoptotic process with less incidence of necrotic death, exhibiting a proinflammatory profile of cytokines characterized by high levels of TNF-α, IL-6, and IL-12, but no IFN-γ. Only SEI induced early apoptosis, suggesting some differences among SAgs kinetic behavior at cell death level. The absence of CD4+ T cells in the culture could explain the lack of IFN-γ production in THP-1 cells. The secretion of this cytokine and the mechanism of cell death in monocytes seem to depend, in part, on the presence of CD4^+^ T cells (76). The released cytokines are in agreement with an M1 profile, with a high inflammatory environment that could promote a Th1 response that it is not the most appropriate for the *S. aureus* eradication. In concordance with these results, no IL-10 was detected, favoring a proinflammatory cytokine storm which is essential for TSS unleashing. It was reported that THP-1 monocyte cells could secrete IL-17A under certain stimuli (77). Nevertheless, not one of the *egc* SAgs induced IL-17A secretion under our experimental conditions.

Monocytic- and macrophagic-like cells showed the same profile of proliferation inhibition and production of proinflammatory cytokines, but only the macrophagic-like cells treated with SEM and SEO had the capacity to induce the production of nitrites at higher concentrations (100 μg/ml, data not shown), a characteristic of classical macrophage activation, which would accompany the previously mentioned inflammatory response.

In order to determine if the effects observed on monocytes were restricted to the action of *egc* SAgs or could be extended to enterotoxins of other bacterial species, we also evaluated the effect of streptococcal superantigen A (SSA) on innate immune system cells. Although staphylococcal and streptococcal SAgs display structural and behavioral similarities, staphylococcal and streptococcal TSS differ in their clinical signs, origin of infection, and prognosis (66, 78). In order to elucidate if *egc* SAgs and SSA shared comparable effects on innate immune system cells, we studied both types of toxins. *egc* SAgs and SSA inhibited monocytic cell proliferation and induced the same monocyte phenotype: SEG, SEI, and SSA induced the overexpression of cluster differentiation molecules 14, 40, and 86. Despite this fact, no increase in phagocytosis was observed in either case, showing an inefficient activation of monocytic cells by these SAgs. In a previous work, we reported that the treatment of dendritic cells with SEG, in the absence of T cells, did not disturb the viability of these cells, increased phagocytosis, and inhibited cell maturation (59).

Monocytic THP-1 cells express high levels of MHC-II molecule DR1, a natural ligand of SAgs (79). The interaction of DR1 with SEG, SEI, and SSA is very well documented (35, 45, 65, 80). Consequently, we investigated if the effect of SAgs on monocytes was mediated by the SAg binding site with DR1. Pre-incubation of SAgs with DR1 avoided the effect of SAgs on monocytic cells. These results could be in concordance with those published by Ferreira Duarte et al. (60) where staphylococcal enterotoxin A (SEA) inhibits mouse neutrophil migration, which is reverted prior to incubation of the cells with an antibody to the MHC-II molecule.

The induction of the expression of CD14 and CD40 after SAg treatment seemed to be independent of the interaction site with the DR1, suggesting that other sites of the SAgs or other molecules on the monocyte surface could be involved in this process. New targets of SAgs on the APC were recently described (42, 81, 82). The interaction between classical SAgs and CD28, and with B7.2 (CD86), the cognate ligand of the CD28, was already described by others (40–44, 47) and could promote a full activation of T cells. The interaction between SAgs and B7.2 would be carried out through a conserved domain among classical and non-classical SAgs. However, the interactions between these new targets and non-classical SAgs have not been studied yet. In our study, we observed that monocytic-like cells treatment with non-classical SAgs induces an overexpression of CD86, which is partially abrogated when SAgs are pre-incubated with the DR1 molecule (Figure 9E). Since macrophages interact with Th1 cells in the periphery, the overexpression of CD86 could promote a strong activation of the Th1 cells, conducting to the depletion of these cells as happens in the lymph node. CD38 was also postulated by Zilber et al. (82) as another SAg ligand on the monocyte surface and could be considered as a possible candidate to cooperate with DR1 and mediate monocytic activation and subsequent death induced by these toxins. Gregory et al. (81) postulated CD1a as a co-stimulatory molecule on monocytes, which could contribute to T cell activation by SAgs.

Only one study reported that a classical staphylococcal superantigen, SEA, could bind to gp130, a signal transductor of IL-6, involving the MHC-II interaction site (48). SEA binds to MHC-II with high affinity, compromising the residues which interact with Zn, promoting a long half-life complex with the beta chain of the DR1 molecule. We previously reported that SEI binds to the β chain of the DR1-MHC-II molecule and the processed peptide in a Zn-dependent manner (45). Since gp130 is expressed on the THP-1 cell surface (73), we evaluated if SEI, a non-classical *egc* SAg, interacts with this protein by SPR. Our results demonstrated that SEI specifically binds to gp130 with moderate affinity and by one order of magnitude lower than the value already reported by SEI-DR1 (*K*_D_ 10^−6^ M). In addition, we observed that the treatment with EDTA, a chelating agent of bivalent cations like Zn, did not abrogate the interaction. We also demonstrated that all *egc* operon non-classical SAgs have specific interaction with gp130, showing a similar kinetic behavior (Figure 10). The physiological role of these interactions could be explained considering that SAgs block gp130, preventing IL-6 stimuli and the activation of STAT-3 pathway, which is needed for the expression of IL-30 in monocytes (73). Since it has been suggested that IL-30 could be involved in the sepsis-control-modulating cytokines secreted by NKT cells (83), inhibition of the synthesis of IL-30 would suit the spread of bacteria. Nevertheless, more studies must be conducted to determine the biological meaning of this interaction.

Considering that neither SEG and SEI nor SSA showed significant differences when human monocytes were treated with them, we could suggest that this first step of infection, which is essential for bacterial dissemination and the subsequent disease prognosis, does not explain the discrepancy of progression to the TSST observed among toxins belonging to different species (66). Nevertheless, it is clear that TSS involves other components of the pathogens which are not evaluated in the present work.

As general conclusions of this work, we propose that SAgs inhibit monocytic/macrophagic effector cells, promoting a partial immunosuppression state of the host in the first stages of the infection, which would favor bacterial spread. Despite that these toxins increase the expression of the differentiation clusters evaluated, which suggest cell activation, they do not stimulate phagocytosis, and as a consequence the viability of the bacteria would not be affected. Furthermore, the high level of proinflammatory cytokines released due to SAgs stimulation could induce a type I acquired immune response. Even if SAgs promote cell death in a high percentage of the monocytic/macrophagic population, the fraction that still remains alive could be maintained primed with SAgs. As a consequence, when Th1 cells contact the SAg-primed macrophages, they would suffer a process of apoptosis or energy, enhancing the immunosuppressive status of the host.

## Data Availability Statement

The datasets generated for this study can be found in the GenBank MK947360, MK947361, MK947362, MK947363, MK947364, MK947365, MK947366.

## Ethics Statement

Animal experiments (antisera production) were reviewed and approved by the Laboratory Animal Welfare Committee CICUAL, Facultad de Farmacia y Bioquimica, Universidad de Buenos Aires, Resolution No: 2349-18.

## Author Contributions

SN, MD, EM, and MMF conceived and designed the experiments and analyzed the data. SN, MD, MS, MA, LI, MJF, and MT performed the experiments. AM and EM contributed with reagents, materials, and analysis tools. SN, MD, EM, and MMF wrote the paper.

### Conflict of Interest

The authors declare that the research was conducted in the absence of any commercial or financial relationships that could be construed as a potential conflict of interest.
