# Supplementary material for: egc Superantigens Impair Monocytes/Macrophages Inducing Cell Death and Inefficient Activation
Source: Front Immunol. 2020 Jan 15;10:3008. doi: 10.3389/fimmu.2019.03008 (PMC6974467; doi:10.3389/fimmu.2019.03008)

Supplementary data

SP Table 1. Specific primers for SAg genes evaluation by PCR

Gen	Primers	Tm (ºC)
tsst1 PR	TTTCCAATAACCACCCGTT	57,35
ttst 1 PF	ATGGCAGCATCAGCTTGATA	54,55
nuc PR	AGCCAAGCCTTGACGAACTAAAGC	63,88
nuc PF	GCGATTGATGGTGATACGGTT	58,79
hsp60 PR	CCATAGCTTCTGAAATATAGCG	54,62
hsp60 PF	GGTTTACGACAAGGTATCGAC	56,48
sea PR	GAACCTTCCCATCAAAAACA	53,96
sea PF	TTGGAAACGGTTAAAACGAA	51,14
seb PR	GCAGGTACTCTATAAGTGCC	55,05
seb PF	TCGCATCAAACTGACAAACG	57,34
sec PR	AAATCGGATTAACATTATCC	49,60
sec PF	GACATAAAAGCTAGGAATTT	48,61
sed PR	TAATGCTATATCTTATAGGG	42,23
sed PF	CTAGTTTGGTAATATCTCCT	46,60
see PR	TAACTTACCGTGGACCCTC	50,00
see PF	TAGATAAAGTTAAAACAAGC	38,10
seg PR	CCAGATTCAAATGCAGAACC	57,77
seg PF	TGCTATCGACACACTACAACC	54,93
seh PR	GACCTTTACTTATTTCGCTGTC	55,23
seh PF	CGAAAGCAGAAGATTTACACG	55,56
sei PR	CCATATTCTTTGCCTTTACCAG	55,13
sei PF	GACAACAAAACTGTCGAAACTG	56,68
sei PR2	GCCCTAGAGACTTTAAAATT	50,37
sej PR	TCTAGCGGAACAACAGTTCTGA	59,37
sej PF	CAGCGATAGCAAAAATGAAACA	56,25
ser PR	TCACATTGTAGTCAGGTGAACTTCT	61,30
ser PF	TGCTATCGACACACTACAACC	59,10

seo PF	AGTCAAGTGTAGACCCTATT	52,64
seo PR	TATGCTCCGAATGAGAATGA	53,95
sem PF	CCAATTGAAGACCACCAAAG	54,76
sem PR	CTTGTCCTGTTCCAGTATCA	54,39
sen PF	ATGAAAAAATTAATAAGCA	52,57
sen PR	TTATATCGTTTCTTCAG	52,53
seu PR	GTGTGACCGAGCATGATGGA	60,11
seu PF	TCGCAGCTATGCAGAACAATC	59,06

SP Table 2. Source and origin of *Staphylococcus aureus* isolates

Strain	Hospitalization area	Outpatient area	Sample	Diagnosis
**40900**	Medical surgery		Abscess	Septic hip loosening
**41026**	Traumatology		Wound	
**41192**	Hematology		Catheter	Febrile neutropenic
**41226**	Triage		Chest injury	
**41395**		Infectology	Abscess	
**41399**	Urology		Abscess	
**41524**	ICU		MiniBAL	Ventilator-associated infection
**41598**	ICU		BAL	Endovascular pathology, ventilator-associated infection.
**41627**	Medical surgery		Blood	Anemic syndrome
**41668**		Traumatology	Knee section	Cellulitis
**41674**	Medical surgery		Drainage	Weapon wound
**41759**	Medical surgery		Blood	
**41762**	Plastic surgery		Fistula	Gluteus silicone prosthesis

BAL: Bronchoalveolar lavage

SP Figure 1. SDS gel of the recombinant *egc* SAgs.

Recombinant *egc* SAgs were purified by affinity and size exclusion chromatography. To evaluate the purity of the recombinant proteins, an electrophoretic chromatography under denaturant conditions (SDS gel) was carried out. The gel was stained with Coomassie blue. As it is showed, one band of proper molecular weight is observed in all cases, except dimmer form for SEO.

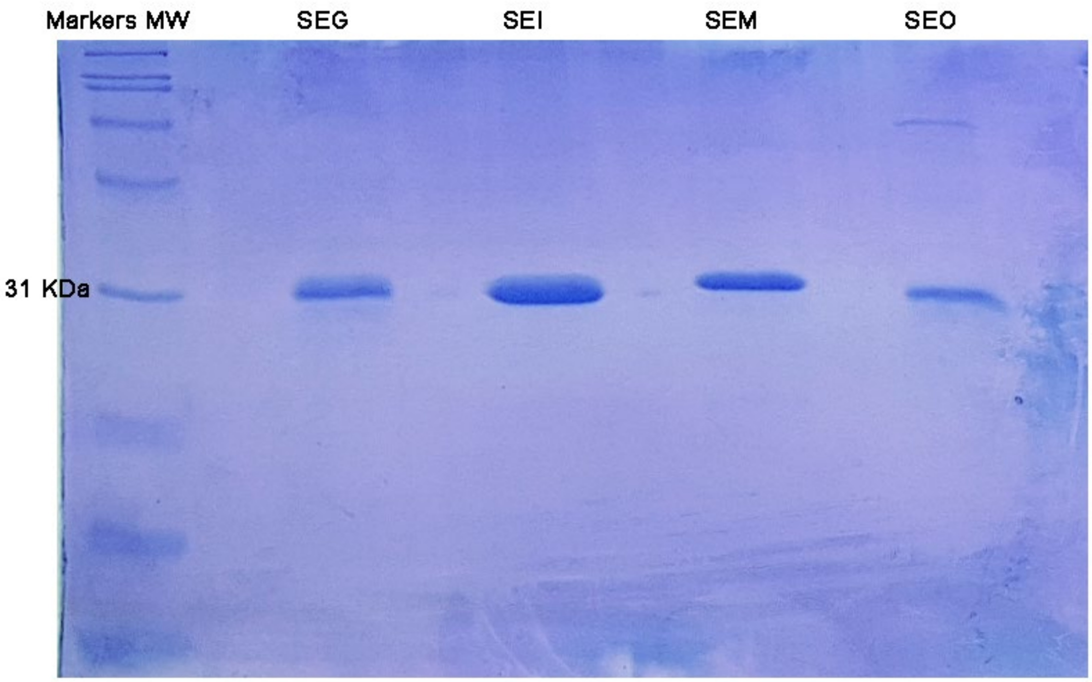

SP Figure 2. Bacteria phagocytosis by THP-1 cells is not affected by SAgs incubation. THP-1 cells were incubated with SAgs for 48h and phagocytosis capacity of fluorescent bacteria was measured by flow cytometry. Data are expressed as the mean ± SEM of at least three independent experiments. Asterisks represent statistical significance with respect to untreated cells within each treatment: ns= non-significant difference.

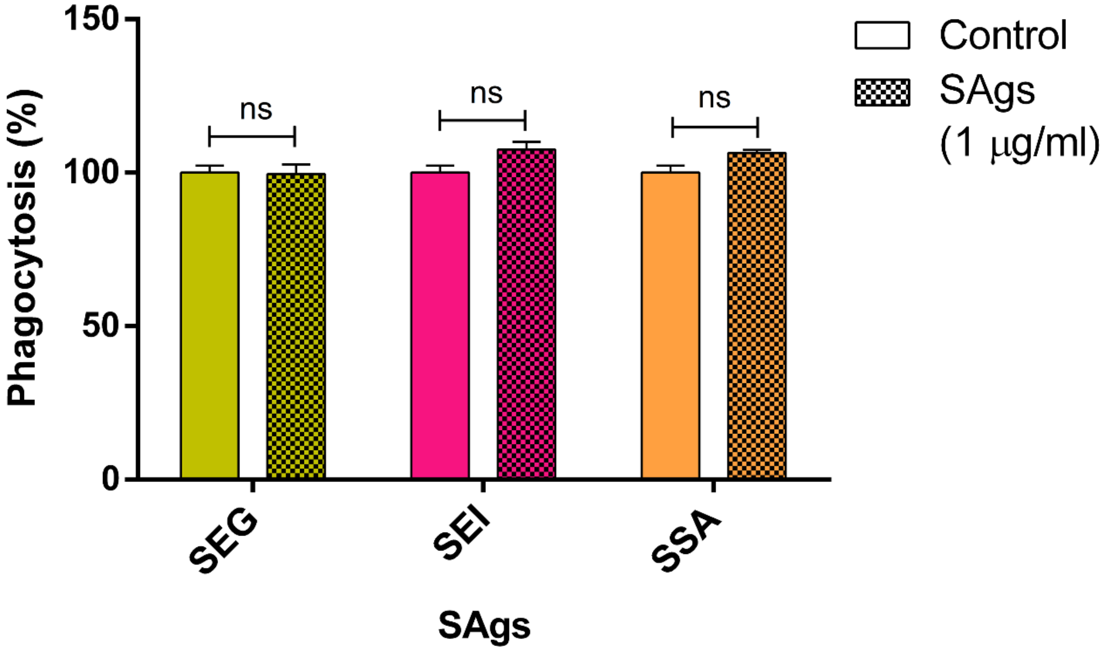

Supplement: Supplementary file 1 [file Data_Sheet_1.pdf]
